# Estimation of split renal function using [^18^F]-flotufolastat PET/CT compared with [^68^Ga]-PSMA-11 and [^99m^Tc]-MAG3 scintigraphy

**DOI:** 10.1186/s13550-026-01385-0

**Published:** 2026-02-03

**Authors:** Michael Christian Marius Gammel, Charlotte Olufs, Kimberley Hansen, Julia Brosch-Lenz, Matthias Heck, Wolfgang A. Weber, Matthias Eiber, Isabel Rauscher

**Affiliations:** 1 Department of Nuclear Medicine, TUM Klinikum, Rechts der Isar, Technical University of Munich (TUM), School of Medicine and Health, Munich, Germany; 2 Department of Urology, TUM Klinikum, Rechts der Isar, Technical University of Munich (TUM), School of Medicine and Health, Munich, Germany; 3 Bavarian Cancer Research Center (BZKF), Erlangen, Germany

**Keywords:** Split renal function, PSMA, [^99m^Tc]Tc-MAG3, Prostate cancer, PET/CT

## Abstract

**Background:**

Physiological prostate-specific membrane antigen (PSMA) expression in renal proximal tubules results in radiopharmaceutical uptake in PSMA-PET, suggesting the potential to assess renal function. To evaluate split renal function derived from [^18^F]F-flotufolastat ([^18^F]F-rhPSMA-7.3) PET/CT and to systematically compare its performance with [^68^Ga]Ga-PSMA-11 PET/CT and the reference standard [^99m^Tc]Tc-MAG3 scintigraphy, we retrospectively analyzed 302 patients with metastatic castration-resistant prostate cancer undergoing PSMA-PET/CT using either [^18^F]F-flotufolastat (*n* = 221) or [^68^Ga]Ga-PSMA-11 (*n* = 81), along with [^99m^Tc]Tc-MAG3 scintigraphy, prior to PSMA radioligand therapy. SRF was calculated from PSMA-PET/CT using mean standardized uptake values and CT-derived renal volumes. SRF was calculated from [^99m^Tc]Tc-MAG3 using standard integral analysis. SRF derived from PSMA-PET/CT ([^18^F]F-flotufolastat and [^68^Ga]Ga-PSMA-11) was correlated with split renal function obtained from [^99m^Tc]Tc-MAG3 scintigraphy on a side-specific basis using Pearson correlation and Bland–Altman analysis. Receiver operating characteristic (ROC) analyses were performed to evaluate diagnostic performance for detecting impaired renal function (SRF ≤ 25% and ≤ 40%). A PET-based accumulation index (ACI) was explored in relation to estimated glomerular filtration rate (eGFR) in an exploratory, tracer-specific analysis.

**Results:**

Strong correlations were found between PET-SRF and [^99m^Tc]Tc-MAG3-SRF (*r* = 0.88 for [^18^F]F-flotufolastat, *r* = 0.85 for [^68^Ga]Ga-PSMA-11; both *p* < 0.0001). Bland–Altman analysis showed a smaller mean bias and narrower limits of agreement for [^18^F]F-flotufolastat (-0.56%; -11.88% to + 10.75%) compared with [^68^Ga]Ga-PSMA-11 (-1.18%; -15.3% to + 12.95%), with 92% versus 83% of values within ± 10%, respectively. ROC analysis confirmed excellent accuracy for identifying [^99m^Tc]Tc-MAG3-SRF ≤ 25% (area under the curve [AUC] = 0.997 for [^18^F]F-flotufolastat; AUC = 0.942 for [^68^Ga]Ga-PSMA-11). No significant association was observed between ACI and eGFR for either radiopharmaceutical ([^18^F]F-flotufolastat: Spearman’s ρ = 0.056, *p* = 0.414; [^68^Ga]Ga-PSMA-11: Spearman’s ρ = − 0.071, *p* = 0.536).

**Conclusion:**

PSMA-PET/CT provides reliable estimates of SRF, with [^18^F]F-flotufolastat showing slightly superior agreement with [^99m^Tc]Tc-MAG3 scintigraphy. This may potentially eliminate the need for additional renal scintigraphy for SRF assessment in the future and may simplify workflows and reduce patient burden.

## Introduction

Prostate cancer is one of the most common malignancies in men [1], requiring precise diagnostic and therapeutic approaches, particularly in advanced stages. Prostate-specific membrane antigen (PSMA) has emerged as a critical target in prostate cancer management due to its overexpression in malignant prostate tissue [2]. Imaging with [^68^Ga]- or [^18^F]-labeled PSMA-targeted radiopharmaceuticals plays a pivotal role in staging, restaging, and monitoring response to therapy [3–9]. Additionally, PSMA-targeted radioligand therapy (RLT) with [^177^Lu]Lutetium-labeled PSMA (LuPSMA) radioligands has become an essential life-prolonging treatment option for patients with metastatic castration-resistant prostate cancer (mCRPC) [10].

Despite its specificity for prostate cancer cells, PSMA is also physiologically expressed in the proximal tubule cells of the kidneys [11–15]. This renal uptake introduces a potential risk of radiation nephrotoxicity during PSMA-RLT [16, 17]. However, its presence also provides an opportunity to evaluate renal function non-invasively using PSMA-PET. The assessment of renal function is critical for prostate patients prior to therapeutic interventions (e.g. external beam radiation therapy, LuPSMA RLT) to minimize toxicity and optimize treatment planning [18].

Until now, dynamic renal scintigraphy using Technetium-99m [^99m^Tc]Tc-mercapto-acetyltriglycine ([^99m^Tc]Tc-MAG3) remains the clinical standard for assessing global and split renal function (SRF) and excluding urinary outflow obstruction. [^99m^Tc]Tc-MAG3 scintigraphy accurately estimates tubular renal function but requires an additional imaging procedure, which can be time-consuming and burdensome, particularly for patients with painful skeletal metastases.

Previous preliminary studies have demonstrated a strong correlation between PSMA-PET-derived SRF and [^99m^Tc]Tc-MAG3-based SRF using [^68^Ga]Ga-PSMA-11 and [^18^F]F-PSMA-1007 [19–21]. However, these studies have been limited by relatively small patient populations (*n* = 28–97). ^18^F- Flotufolastat ([^18^F]F-rhPSMA-7.3), is a recently FDA-approved radiohybrid PSMA-PET radiopharmaceutical for staging and restaging of prostate cancer [22, 23] but has not yet been investigated in assessing renal function.

In this study, we retrospectively evaluated a large cohort of patients with mCRPC who underwent both PSMA-PET and [^99m^Tc]Tc-MAG3 scintigraphy. We aimed to compare the accuracy of SRF derived using [^68^Ga]Ga-PSMA-11 and [^18^F]F-flotufolastat versus the standard [^99m^Tc]Tc-MAG3 scintigraphy. Additionally, we investigated correlations between absolute renal uptake derived from PSMA-PET, tubular excretion rate (TER) of [^99m^Tc]Tc-MAG3, and serum creatinine levels to explore global renal function parameters.

## Materials and methods

### Patient characteristics

This retrospective study included 302 patients with advanced mCRPC who underwent either [^68^Ga]Ga-PSMA-11 PET/CT (*n* = 81) or [^18^F]F-flotufolastat (*n* = 221) and [^99m^Tc]Tc-MAG3 scintigraphy as part of their clinical evaluation for PSMA RLT at the, Technical University of Munich University Hospital, Germany. All patients provided written informed consent for the clinical examination and reported investigations were conducted in accordance with the Declaration of Helsinki and with national regulations. The local ethics committee approved the retrospective analysis (permits: 2019-99_2-S-SR, updated on May 31, 2023). All patients evaluated for RLT between October 2014 and October 2022 were included, with a median interval of 15 days between PSMA-PET and [^99m^Tc]Tc-MAG3 scintigraphy. Patient characteristics are summarized in Table 1.

**Table 1:** Patient characteristics

Characteristic	All patients (*N* = 302)
Patients who underwent [^68^Ga]Ga-PSMA-11 PET/CT, n	81
Patients who underwent [^18^F]F-flotufolastat PET/CT, n	221
Age, years	72 ± 10
Injected activity [^18^F]F-flotufolastat, MBq	288 ± 60
Injected activity [^68^Ga]Ga-PSMA-11, MBq	122 ± 36
Acquisition p.i. [^18^F], minutes	72 ± 13
Acquisition p.i. [^68^Ga], minutes	56 ± 12
TER, mL/min	201 ± 46
Creatinine, mg/dL	1.0 ± 0.29
eGFR, mL/min/1.73 m^2^	77 ± 19
Patients with eGFR > 60 ml/min, n	228
Patients with eGFR ≤ 60 ml/min, n	74
Split function, %	50 ± 12
PSA_mean_, ng/mL	338 ± 700

Unless otherwise stated, all values are mean ± SD

^68^Ga = Gallium-68; PSMA = prostate-specific membrane antigen; PET = positron emission tomography; CT = computed tomography; ^18^F = Fluorine-18; rh = radiohybrid; MBq = megabecquerel; TER = tubular excretion rate; eGFR = estimated glomerular filtration rate, PSA = prostate-specific antigen

### [^18^F]F-flotufolastat PET/CT and [^68^Ga]Ga-PSMA-11 PET/CT

The radiolabeling of both [^18^F]F-flotufolastat and [^68^Ga]Ga-PSMA-11 followed established protocols described in previous studies [24, 25].

For [^18^F]F-flotufolastat, an intravenous bolus of 288 ± 60 MBq was administered, and PET scanning commenced approximately 72 ± 13 min post injection. For [^68^Ga]Ga-PSMA-11, a mean activity of 122 ± 36 MBq was injected intravenously, with PET scanning commencing at a mean of 56 ± 12 min post-injection. All patients received a diluted oral contrast medium (300 mg Telebrix, Guerbet) and a diagnostic CT scan in the portal venous phase, performed 80 s after intravenous administration of iodinated contrast (Imeron 300, Bracco Imaging). PET/CT scans for both [^18^F]F-flotufolastat and [^68^Ga]Ga-PSMA-11were conducted using either a Siemens Biograph mCT Flow or Siemens Biograph Vision 600 scanner (Siemens Healthineers. Imaging was performed in 3D mode, with acquisition speeds of 0.8 mm/s (Biograph mCT Flow) and 1.1 mm/s (Biograph Vision 600) for [^18^F]F-flotufolastat, and 1.1–1.5 mm/second or 3–4 min per bed position for [^68^Ga]Ga-PSMA-11.

Reconstruction was carried out using ordered-subset expectation maximization (TrueX, 4 iterations, 8 subsets) for both radiopharmaceuticals. A Gaussian smoothing filter of 2 mm (full width at half maximum) was applied for [^18^F]F-flotufolastat, and 5 mm (full width half maximum) for [^68^Ga]Ga-PSMA-11. Emission data were corrected for randoms, dead time, scatter, and attenuation for both protocols, ensuring consistent and accurate quantitative analysis.

### SRF from PSMA-PET

SRF was calculated using PET data based on renal uptake of regions of interest (ROI) were delineated using 50% isocontour thresholds to extract both maximum standardized uptake value (SUV_max_) and mean standardized uptake value (SUV_mean_). For PET, ROIs were manually drawn over the kidneys, excluding the renal pelvic caliceal system (RPCS). On CT, the renal parenchyma was defined while excluding the renal pelvic caliceal system and cystic structures.

The SRF was calculated using the following formula:

$$\begin{aligned}&\:{\mathrm{SRF}}_{\mathrm{right}}\\&=\frac{{\mathrm{Volume}}_{\mathrm{right}}\times\:{\mathrm{SUV}}_{\mathrm{right}}}{{\mathrm{Volume}}_{\mathrm{right}}\times\:{\mathrm{SUV}}_{\mathrm{right}}+{\mathrm{Volume}}_{\mathrm{left}}\times\:{\mathrm{SUV}}_{\mathrm{left}}}\end{aligned}$$

Calculations were performed separately using SUV_max_ and SUV_mean_, with volumes derived from either PET (functional) or CT (anatomical) data. All ROI placements and analyses were reviewed by an experienced nuclear medicine physician (I.R.) to ensure consistency and accuracy.

### Calculation and statistical evaluation of the PET-Based accumulation index (ACI)

To explore potential associations between PSMA-PET-derived measures and global renal function, an Accumulation Index (ACI) was calculated for each patient by dividing the combined renal parenchymal volume (segmented from CT) by the sum of the SUV_mean_ estimates from both kidneys. This approach was inspired by the methodology proposed by Weissinger et al. for somatostatin receptor targeted PET/CT imaging, where the ACI showed strong positive correlation with MAG3-derived renal function parameters [26]. Although PSMA tracers differ substantially in their pharmacokinetics, particularly regarding proximal tubular binding, we considered this concept worth exploring in our cohort. Due to non-normal distribution of ACI and estimated glomerular filtration rate (eGFR) (Shapiro–Wilk test, *p* < 0.0001 for both), correlations were assessed using Spearman’s rank correlation coefficient (ρ). Analyses were performed separately for [^18^F]F-flotufolastat and [^68^Ga]Ga-PSMA-11.

### [^99m^Tc]Tc-MAG3-Scintigraphy

To ensure optimal hydration, all patients were instructed to drink at least 10 mL of mineral water per kilogram of bodyweight 30 min prior to scintigraphy. Dynamic renal scintigraphy with [^99m^Tc]Tc-MAG3 was performed per European Association of Nuclear Medicine guidelines [27] and the Bubeck method [28], using approximately 100 MBq of [^99m^Tc]Tc-MAG3 and planar dynamic imaging with both posterior and anterior detectors for 20 min. Current CT images were used to determine whether calculation of the geometric mean was required. ROI were drawn manually over the renal parenchyma, including the renal pelvic caliceal system, as well as the aorta and background regions. In cases of inadequate[^99m^Tc]Tc-MAG3 excretion observed in post-micturition images, furosemide was administered, followed by an additional 20 min of imaging. SRF was calculated from renal activity curves between 60 and 100 s. Tubular extraction rate (TER) was determined using the two-sample plasma clearance method, and side-separated TER was calculated by multiplying SRF with the total TER.

### Statistical analysis

All statistical analyses were performed using Microsoft Excel and MedCalc version 23.1.3 (MedCalc Software Ltd, Ostend, Belgium). Continuous variables were expressed as mean ± standard deviation. Normality was assessed using the Shapiro–Wilk test. The Pearson correlation coefficient (r) was calculated to evaluate the linear relationship between PSMA-PET-derived SRF(PET-SRF) and [^99m^Tc]Tc-MAG3-derived SRF ([^99m^Tc]Tc-MAG3-SRF) as the reference standard. In addition, correlations between PET-based measures of absolute renal uptake and TER were assessed to evaluate global renal function. Bland–Altman analysis [29, 30] was conducted to assess agreement between methods, including the calculation of mean differences, limits of agreement, and the proportion of values within a predefined range of ± 10%. Linear regression analysis was performed to model the relationship between PET-SRF and [^99m^Tc]Tc-MAG3-SRF, with coefficients of determination (R^2^), regression equations, and residual analyses reported. Normality of residuals was tested using the D’Agostino–Pearson test.

Receiver operating characteristic (ROC) curve analysis was conducted to evaluate the diagnostic accuracy of PET-SRF for identifying severely impaired renal function ([^99m^Tc]Tc-MAG3-SRF ≤ 25%) and reduced renal function ([^99m^Tc]Tc-MAG3-SRF ≤ 40%). The area under the ROC curve (AUC), sensitivity, specificity, and optimal thresholds based on the Youden Index were reported. Statistical significance was set at *p* < 0.05.

## Results

Renal function parameters were within an overall preserved range, with a mean serum creatinine of 1.0 ± 0.29 mg/dL and mean eGFR of 77 ± 19 mL/min/1.73 m². A total of 228 (75%) patients presented with an eGFR above 60 mL/min. Mean TER was 201 ± 46 mL/min. Mean SRF based on [^99m^Tc]Tc-MAG3 was 50 ± 12%.

### SRF

**Fig. 1:**
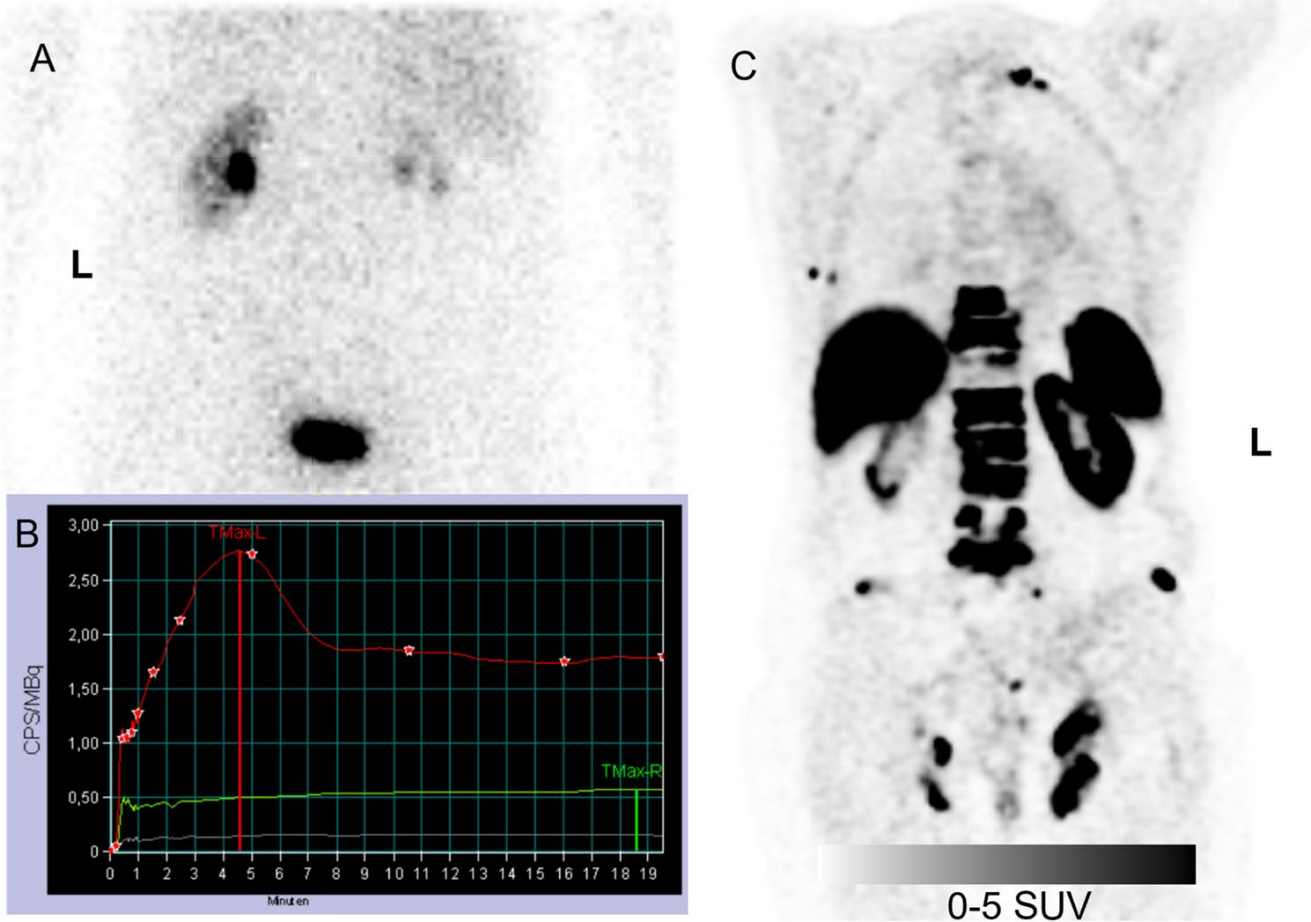
(**A**) [^99m^Tc]Tc-MAG3 scintigraphy acquired 20 min post-injection after administration of 112 MBq, with the corresponding renogram curves (**B**) of both kidneys shown below. (**C**) Coronal PET/CT images acquired 62 min post-injection after administration of 340 MBq [^18^F]F-flotufolastat are shown on the right. Split renal function calculated from both modalities shows concordant results, with MAG3 scintigraphy indicating a functional contribution of 94% for the left kidney and 6% for the right kidney, and PET-based analysis showing 91% and 9%, respectively ^18^F = Fluorine-18; MAG3 = mercapto-acetyltriglycine; PET = positron emission tomography; SRF = split renal function; ⁹⁹ᵐTc = Technetium-99 m

*Correlation Between PET- and* [^99m^Tc]Tc-MAG3*-Derived SRF* A strong linear relationship was observed across all tested PET SRF combinations (SUV_max_ and SUV_mean_, volumes derived from PET or CT) and [^99m^Tc]Tc-MAG3-SRF, with Pearson correlation coefficients (r) ranging from 0.835 to 0.881 (all *p* < 0.0001). The best agreement was achieved using SUV_mean_ in combination with CT-based renal volumes (*r* = 0.881), therefore all subsequent analyses were performed using this approach. An illustrative example is provided in Fig. 1. Table 2 summarizes the findings of the ¹⁸F-flotufolastat analysis.

**Table 2:** Methods for SRF calculation using ¹⁸F-flotufolastat

Methods for SRF-calculation using ¹⁸F-flotufolastat	Pearson *r*	*p*-value
[^99m^Tc]Tc-MAG3**-**SRF & SRF(SUV_mean_, CT-Volume)	0.881	< 0.0001
[^99m^Tc]Tc-MAG3**-**SRF & SRF(SUV_max_, CT-Volume)	0.878	< 0.0001
[^99m^Tc]Tc-MAG3**-**SRF & SRF(SUV_max_, PET-Volume)	0.849	< 0.0001
[^99m^Tc]Tc-MAG3**-**SRF & SRF(SUV_mean_, PET-Volume)	0.835	< 0.0001

SRF = split renal function; ^18^F = Fluorine-18; rh = radiohybrid; PSMA = prostate-specific membrane antigen; MAG3 = mercapto-acetyltriglycine, SUV_mean_ = mean standardized uptake value; CT = computed tomography; SUV_max_ = maximum standardized uptake value; PET = positron emission tomography; ⁹⁹ᵐTc = Technetium-99 m

**Fig. 2:**
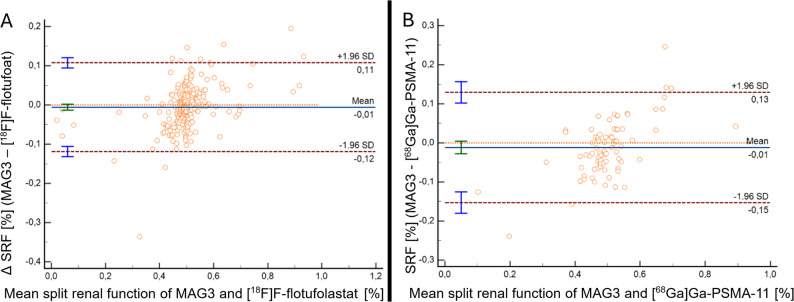
Bland–Altman plots comparing PSMA-PET-derived SRF with [^99m^Tc]Tc-MAG3-based SRF, showing (**A**) agreement between [^18^F]F-flotufolastat -derived SRF and [^99m^Tc]Tc-MAG3-derived SRF, and (**B**) agreement between [^68^Ga]Ga-PSMA-11-derived SRF and [^99m^Tc]Tc-MAG3-derived SRF Solid blue lines represent mean differences, while dashed brown lines indicate the limits of agreement (± 1.96 SD) ^18^F = Fluorine-18; ^68^Ga = Gallium-68; MAG3 = mercapto-acetyltriglycine; PET = positron emission tomography; SD = standard deviation; SRF = split renal function; ⁹⁹ᵐTc = Technetium-99 m

*Agreement Between PET- and* [^99m^Tc]Tc-MAG3*-SRF Estimates* Bland–Altman analysis revealed no significant systematic bias for either radiopharmaceutical. For [^68^Ga]Ga-PSMA-11, the mean difference between PET-SRF and [^99m^Tc]Tc-MAG3-SRF was − 1.18% (*p* = 0.15), with limits of agreement ranging from − 15.30% to 12.95%. Additionally, 83% of values were within ± 10% of agreement. For [^18^F]F-flotufolastat, the mean difference was slightly smaller at −0.56% (*p* = 0.15), with narrower limits of agreement (−11.88% to + 10.75%) and a higher proportion of values (92%) falling within ± 10%. (Fig. 2)

**Fig. 3:**
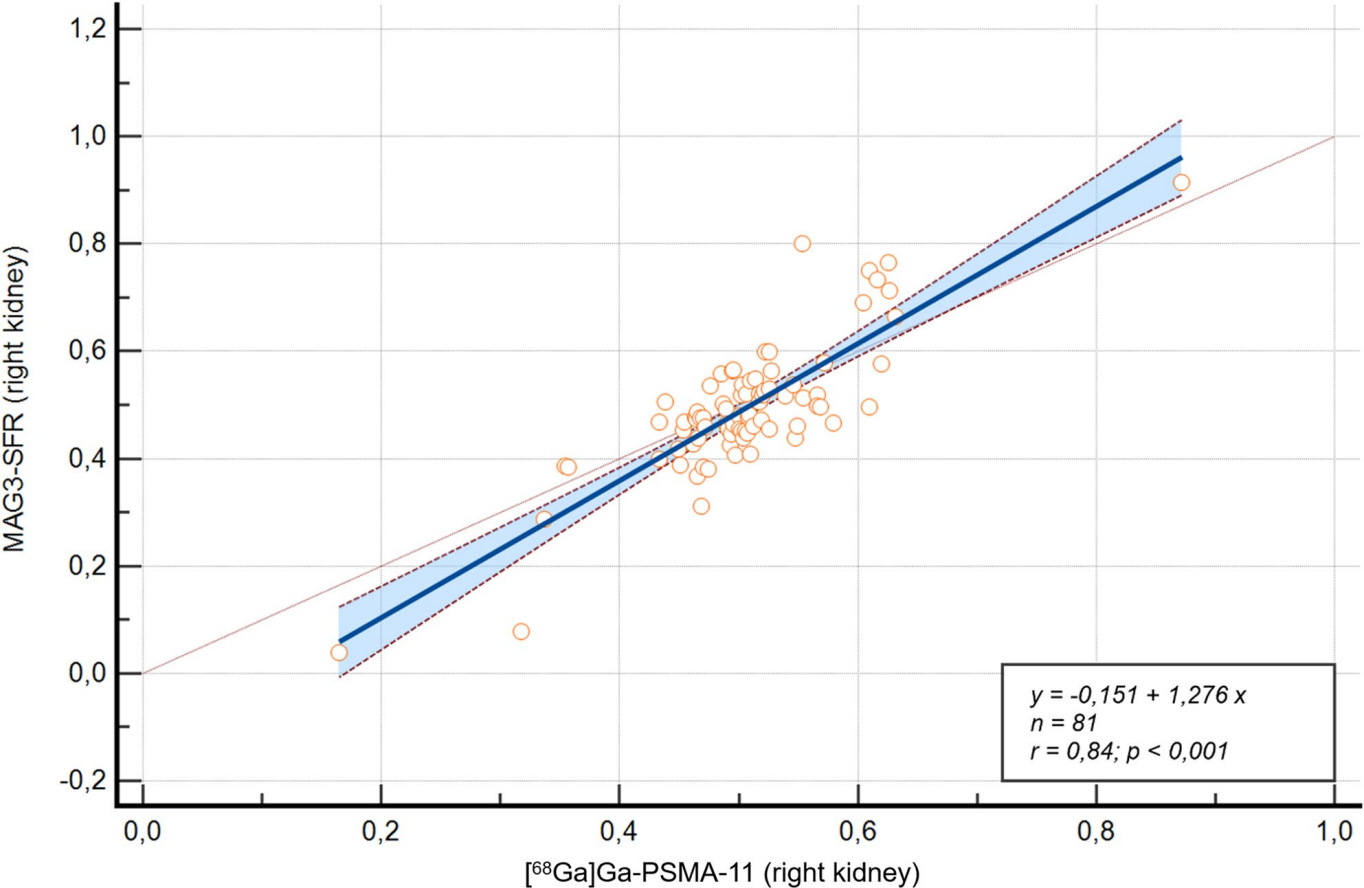
Correlation between [^68^Ga]Ga-PSMA-11-based SRF and [^99m^Tc]Tc-MAG3-derived SRF for the right kidney ^68^Ga = Gallium-68; MAG3 = mercapto-acetyltriglycine; PET = positron emission tomography; SRF = split renal function; ⁹⁹ᵐTc = Technetium-99 m

*Modeling the Relationship Between PET- and* [^99m^Tc]Tc-MAG3*-SRF* The relationship between PET-SRF and [^99m^Tc]Tc-MAG3-SRF was modeled using linear regression. For [^68^Ga]Ga-PSMA-11, the linear regression model produced an R^2^-value of 0.703, with the equation y = − 0.1513 + 1.2763x (Fig. 3). The slope (β1 = 1.2763) significantly deviated from 1 (*p* < 0.0001), suggesting a slight overestimation of higher SRF values by PET.

**Fig. 4:**
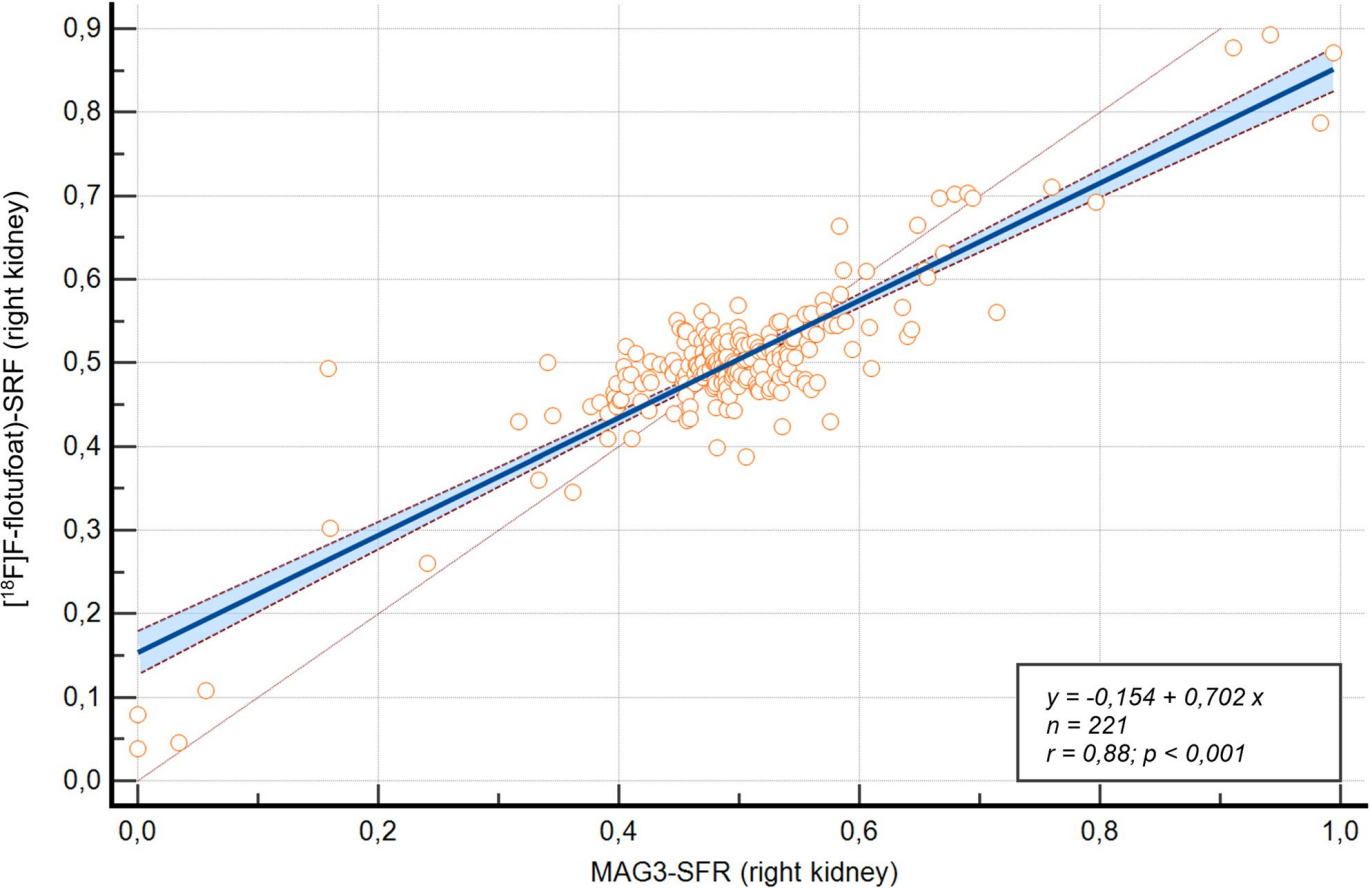
Correlation between [^18^F]F-flotufolastat-based SRF and [^99m^Tc]Tc-MAG3-derived SRF for the right kidney ^18^F = Fluorine-18; MAG3 = mercapto-acetyltriglycine; PET = positron emission tomography; SRF = split renal function; ⁹⁹ᵐTc = Technetium-99 m

By comparison, [^18^F]F-flotufolastat showed a stronger fit, with an R^2^-value of 0.7738 and the regression equation y = 0.1536 + 0.7020x (Fig. 4). The slope (β1 = 0.7020) also significantly deviated from 1 (*p* < 0.0001), indicating a slight underestimation. However, residuals for [^18^F]F-flotufolastat were not normally distributed (*p* < 0.0001, D’Agostino–Pearson test), potentially limiting the model’s validity for extreme values. An overview of both regression models is provided in Table 3.

**Table 3:** Linear regression models for estimating [^99m^Tc]Tc-MAG3-SRF from PET-SRF values

Radioligand	*R*²	Regression equation	Slope (β₁) ≠ 1 (*p*)	Intercept (β₀)	Residuals normally distributed (*p*)
**⁶⁸Ga-PSMA-11**	0.703	*y* = − 0.1513 + 1.2763x	< 0.0001	−0.1513	yes (*p* > 0.05)
**¹⁸F-flotufolastat**	0.774	*y* = 0.1536 + 0.7020x	< 0.0001	0.1536	no (*p* < 0.0001)

MAG3 = mercapto-acetyltriglycine; SRF = split renal function; PET = positron emission tomography; R^2^ = coefficient of determination; ^68^Ga = Gallium-68; PSMA = prostate-specific membrane antigen; ^18^F = Fluorine-18; ⁹⁹ᵐTc = Technetium-99 m

*Diagnostic Accuracy of PET-SRF to Detect Functional Impairment* The ROC analysis confirmed the excellent diagnostic performance of both [^68^Ga]Ga-PSMA-11 and [^18^F]F-flotufolastat in distinguishing impaired split renal function at thresholds of [^99m^Tc]Tc-MAG3-SRF ≤ 25% and ≤ 40%.

At the 25% threshold, [^68^Ga]Ga-PSMA-11 achieved an AUC of 0.942 (95% CI: 0.866–0.982; *p* < 0.0001), with a sensitivity of 84% and a specificity of 100%. [^18^F]F-flotufolastat demonstrated a slightly superior performance with an AUC of 0.997 (95% CI: 0.978–1.000; *p* < 0.0001), achieving a sensitivity of 98% and a specificity of 100%.

**Fig. 5:**
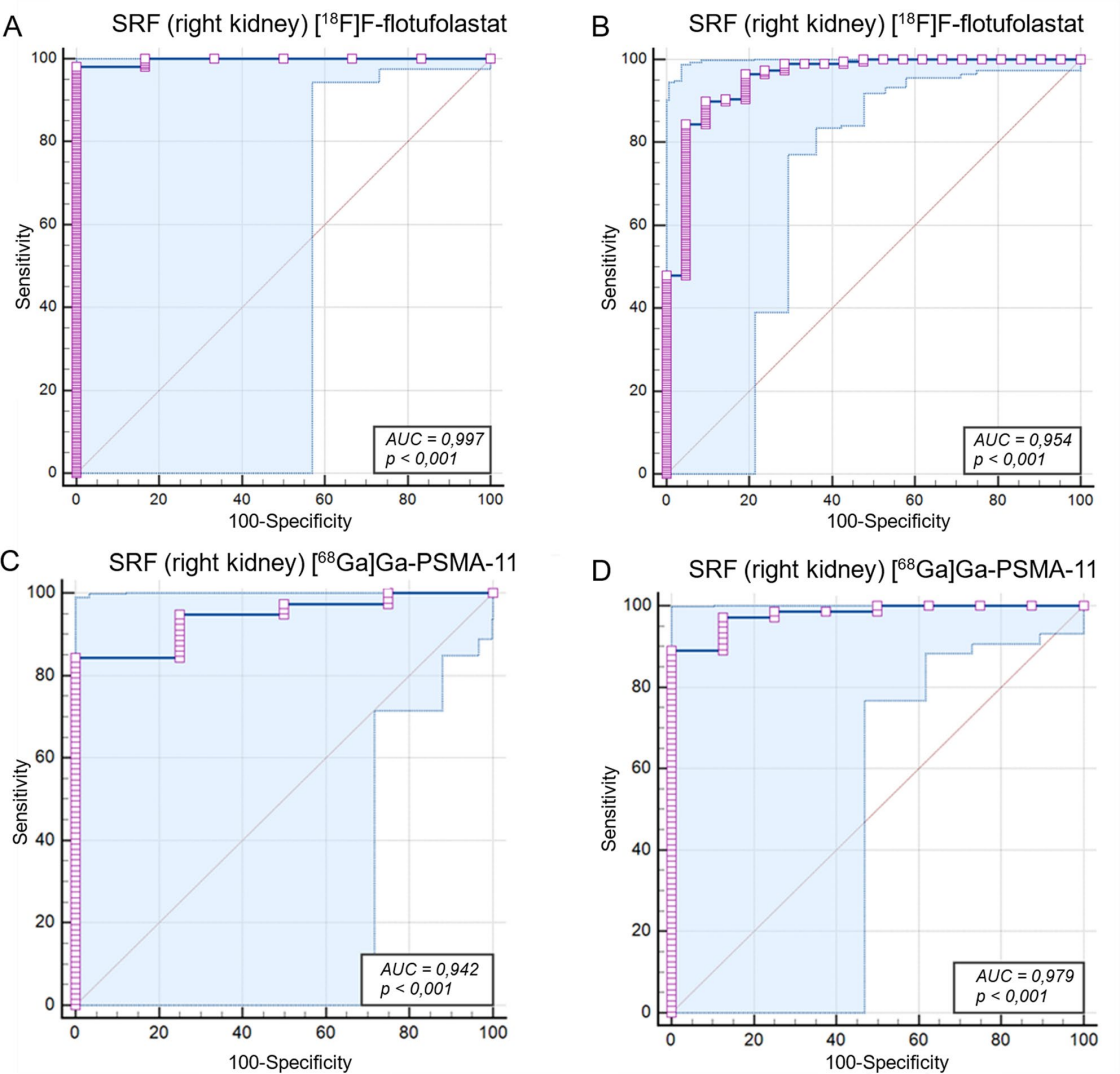
ROC curves for (**A**) [^18^F]F-flotufolastat PSMA-PET-derived SRF in identifying [^99m^Tc]Tc-MAG3-SRF < 25%; (**B**) [^18^F]F-flotufolastat-derived SRF in identifying [^99m^Tc]Tc-MAG3-SRF < 40%; (**C**) [^68^Ga]Ga-PSMA-11-derived SRF in identifying [^99m^Tc]Tc-MAG3-SRF < 25%; and (**D**) [^68^Ga]Ga-PSMA-11-PSMA-PET-derived SRF in identifying [^99m^Tc]Tc-MAG3-SRF < 40% ^18^F = Fluorine-18; ^68^Ga = Gallium-68; AUC = area under the ROC curve; MAG3 = mercapto-acetyltriglycine; PET = positron emission tomography; PSMA = prostate-specific membrane antigen; ROC = receiver operating characteristic; SRF = split renal function; ⁹⁹ᵐTc = Technetium-99 m

At the 40% threshold, [^68^Ga]Ga-PSMA-11 showed an AUC of 0.979 (95% CI: 0.920–0.998; *p* < 0.0001), with a sensitivity of 89% and a specificity of 100%. Similarly, [^18^F]F-flotufolastat achieved an AUC of 0.954 (95% CI: 0.917–0.978; *p* < 0.0001), with a sensitivity of 90% and a specificity of 90%. (Fig. 5)

### Assessment of global renal function using the PET-Based ACI

The ACI showed a non-normal distribution with pronounced right skew and kurtosis (Shapiro–Wilk *p* < 0.0001). The mean eGFR in the overall cohort was 77 ± 19 mL/min/1.73 m². For patients who underwent [^18^F]F-flotufolastat PET/CT (*n* = 212), no significant correlation was observed between ACI and eGFR (Spearman’s ρ = 0.056 [*p* = 0.414]). Similarly, in the [^68^Ga]Ga-PSMA-11 subgroup (*n* = 79), no significant association was found (Spearman’s ρ = − 0.071 [*p* = 0.536]).

## Discussion

Renal function is a critical factor for many prostate cancer therapies, including [^177^Lu]Lu-PSMA therapy, and PSMA-PET/CT offers the potential advantage of calculating SRF as part of every routine scan. Previous studies have explored various radiopharmaceuticals in small cohorts as proof-of-concept investigations (*n* = 28–97) [19–21]. To the best of our knowledge, this is the first study to directly compare [^18^F]F-flotufolastat with [^68^Ga]Ga-PSMA-11 against [^99m^Tc]Tc-MAG3 renal scintigraphy, and to do so in a large patient cohort. This study demonstrated a strong correlation (see Table 2) between PSMA-PET-derived SRF and [^99m^Tc]Tc-MAG3-based SRF for both radiopharmaceuticals, with [^18^F]F-flotufolastat exhibiting a slightly superior diagnostic performance due to its narrower limits of agreement and higher sensitivity.

The strong correlations with [^99m^Tc]Tc-MAG3 observed in this study (*r* = 0.85 for [^68^Ga]Ga-PSMA-11 and *r* = 0.88 for [^18^F]F-flotufolastat) are in line with prior PSMA PET studies, which reported correlation coefficients between *r* = 0.85 and *r* = 0.96 for SRF estimation compared to MAG3 scintigraphy. For example, Rosar et al. found *r* = 0.91 for [^68^Ga]Ga-PSMA-11, Gabela et al. reported *r* = 0.96 for the same tracer, and Rassek et al. demonstrated *r* = 0.87 for [^18^F]F-PSMA-1007 [31–34]. Notably, [^99m^Tc]Tc-DMSA scintigraphy achieves similar correlation coefficients for SRF when compared to MAG3 or DTPA-based methods, typically in the range of *r* = 0.82–0.99 [35–38]. A compact overview of SRF estimation across imaging techniques in literature in comparison to our results is provided in Table 4.

**Table 4:** Correlation coefficients for different renal function assessment methods

Imaging method 1	Imaging method 2	Correlation range (*r*)	References
Renal catheter	DTPA	0.94	[31, 32]
DMSA	DTPA	0.83–0.99	[33, 34]
[^99m^Tc]Tc-MAG3	DMSA	0.82–0.96	[35–38]
[^18^F]F-PSMA-1007	[^99m^Tc]Tc-MAG3	0.87	[19]
[^68^Ga]Ga-PSMA-11	[^99m^Tc]Tc-MAG3	0.91–0.96	[20, 21]
[^68^Ga]Ga-PSMA-11	[^99m^Tc]Tc-MAG3	0.85	Present study
[^18^F]F-rhPSMA-7.3	[^99m^Tc]Tc-MAG3	0.88	Present study

DTPA =diethylenetriaminepentaacetic acid, DMSA = 2,3 dimercaptosuccinic acid, MAG3 = mercapto-acetyltriglycine, ^68^Ga = Gallium-68; PSMA = prostate-specific membrane antigen; ^18^F = Fluorine-18, rh = radiohybrid

Besides its use in primary staging or restaging in biochemical recurrent disease, PSMA-PET/CT is frequently utilized to monitor response in patients with advanced, metastatic prostate cancer under systemic treatment involving nephrotoxic agents, such as PSMA-RLT [7–9]. In addition to assessing global renal function through laboratory parameters, PSMA-PET/CT may provide side-separated renal function data as an inherent component of the imaging process, enabling clinicians to monitor changes in individual kidneys during treatment.

Another possible application might be palliative external beam radiation therapy planning of retroperitoneal and/or lumbar spine lesions, where precise assessment of SRF is critical to minimize renal radiation exposure. By deriving SRF directly from PSMA-PET/CT, the need for additional imaging (such as [^99m^Tc]Tc-MAG3 scintigraphy) may be avoided, reducing procedural complexity for patients with advanced disease who often require optimized and streamlined care.

In general, urinary tract obstruction is a relevant concern in patients with metastatic prostate cancer. Already at the time of initial diagnosis, hydronephrosis is present in approximately 23% of patients with newly diagnosed metastatic prostate cancer, and an additional 21% develop hydronephrosis during the course of their disease, as observed in a small retrospective cohort of 48 patients [39]. Such cases often require interventions like ureteral stenting or nephrostomy prior to systemic therapy. In this context, diuretic renal scintigraphy remains the gold standard for evaluating urinary outflow: with modern 3D dynamic Cadmium-Zink-Tellurid Single-Photon Emission Computed Tomography (CZT SPECT/CT) imaging, it can achieve a sensitivity of 100% and a specificity of 93% in detecting acute ureteric obstruction, as shown by Ochoa-Figueroa et al. in a recent prospective study [40]. Future research could explore the potential of dynamic PSMA-PET/CT to assess renal clearance and identify obstructive uropathies, similar to the capabilities of dynamic [^99m^Tc]Tc-MAG3 scintigraphy. Additionally, longitudinal studies at multiple time points, such as before therapy initiation and after each PSMA-RLT cycle, might provide valuable insights into renal function changes over time. These advancements could help establish PSMA-PET/CT as a more comprehensive tool for both functional and anatomical renal assessment.

The main limitation of this study is the inherent patient cohort, as it consists of individuals with relatively preserved renal function who were eligible for PSMA-RLT. Patients with severe renal impairment are not candidates for PSMA-RLT and were therefore underrepresented in this study. While this may limit statistical accuracy at extreme SRF values, this does not diminish the clinical relevance, as the method is designed for application in a similar patient population where renal function is generally sufficient for PSMA-based treatments.

## Conclusion

PSMA-PET/CT reliably assesses split renal function with strong agreement to [^99m^Tc]Tc-MAG3 scintigraphy, particularly with [^18^F]F-flotufolastat showing slightly superior diagnostic accuracy. This may potentially eliminate the need for additional renal scintigraphy in the future and may simplify workflows and reduce patient burden. Future studies focusing on dynamic PET/CT and longitudinal renal monitoring could further enhance its utility, reinforcing its role as a comprehensive “one-stop-shop” imaging solution for patients with prostate can

## Data Availability

The datasets used and/or analysed during the current study are available from the corresponding author on reasonable request.

## Abbreviations

PSMA: Prostate-specific membrane antigen
PET: Positron emission tomography
CT: Computed tomography
SRF: Split Renal Function
[^99^mTc]Tc-MAG3: Technetium-99 m Mercaptoacetyltriglycine
[^18^F]F-rhPSMA-7.3: Fluor-18 Flotufolastat (radiohybrid PSMA ligand)
[^68^Ga]Ga-PSMA-11: Gallium-68 PSMA-11
ROC: Receiver operating characteristic
ACI: Accumulation index
eGFR: Estimated glomerular filtration rate
AUC: Area under the curve
RLT: Radioligand therapy
LuPSMA: Lutetium-177–labeled PSMA radioligand
mCRPC: Metastatic castration-resistant prostate cancer
TER: Tubular extraction rate
ROI: Region of interest
SUVmax: Maximum standardized uptake value
SUVmean: Mean standardized uptake value
RPCS: Renal pelvic caliceal system
DMSA: Dimercaptosuccinic acid
DTPA: Diethylenetriaminepentaacetic acid
CZT: Cadmium-zinc-telluride
SPECT: Single photon emission computed tomography
R²: Coefficient of determination
CI: Confidence interval
